# Structure-properties relationships in triarylamine-based donor-acceptor molecules containing naphtyl groups as donor material for organic solar cells

**DOI:** 10.1038/srep09031

**Published:** 2015-03-12

**Authors:** Salma Mohamed, Dora Demeter, Jean-Alex Laffitte, Philippe Blanchard, Jean Roncali

**Affiliations:** 1 Group Linear Conjugated Systems, CNRS UMR 6200, MOLTECH-Anjou, University of Angers 2 Bd Lavoisier, 49045 Angers, France; 2 ARKEMA, groupement de recherche de Lacq, Po Box 34 RN 117, 64170 Lacq, France

## Abstract

The effects of replacing the phenyl rings of triphenylamine (TPA) by naphtyl groups are analysed on a series of push-pull molecules containing a 2-thienyl-dicyanovinyl acceptor group. UV-Vis absorption spectroscopy and cyclic voltammetry show that the introduction of one or two naphtyl groups in the structure has limited effects on the optical properties and energy levels of the molecule. On the other hand, the evaluation of the compounds as donor material in bi-layer solar cells with C_60_ as acceptor shows that the number and mode of linkage of the naphtyl groups exert a marked influence on the power conversion efficiency (*PCE*) of the cell. Two naphtyl groups lead to a decrease of *PCE* with respect to TPA, while a single naphtyl group produces opposite effects depending on the linking mode. Compared to TPA, an alpha-naphtyl group leads to a small decrease of *PCE* while in contrast a beta-naphtyl leads to a ~35% increase of *PCE* due to improved short-circuit current density (*J_sc_*) and fill-factor. The determination of the hole-mobility of these two donors by the space-charge-limited current method shows that these effects are correlated with the higher hole-mobility of the β-naphtyl compound.

The chemistry of active materials for organic photovoltaics (OPV) is a highly active field of research powered by the exciting technological opportunities offered by the lightness, plasticity and flexibility of organic materials^1^,^2^,^3^,^4^,^5^,^6^,^7^,^8^,^9^. Besides innovative potential applications, the major motivation for developing OPV remains an expected drastic reduction of the cost and environmental impact of the production of solar cells compared to the established silicon technology. Although soluble conjugated polymers remain a major class of donor materials for solution-processed bulk heterojunction OPV cells^1^,^2^,^3^,^4^, recent years have seen the emergence of small molecules on the forefront of research on OPV materials owing to the obvious advantages of well-defined chemical structures in terms of reproducibility of synthesis, purification, composition and properties of the materials combined with the possibility to develop more reliable analyses of structure-properties relationships.^5^,^6^,^7^,^8^,^9^ After seminal work published in 2005^10^ the design of soluble molecular donors has generated considerable research efforts^5^,^6^,^7^,^8^,^9^,^10^,^11^,^12^,^13^ and solution-processed bulk heterojunction (BHJ) cells with power conversion efficiency (*PCE*) of 8.0–10% have been reported^11^,^12^,^13^. However, these results have been obtained with fully optimized solar cells of small active areas and with donor materials of relatively complex chemical structure prepared by multi-step syntheses which can pose the problem of the overall yield, cost environmental impact and scalability of the active material. As discussed in some recent reviews, one of the key for a future industrial development of OPV technology lies in the drastic reduction of the cost and environmental impact of the synthesis of active materials^14^,^15^,^16^. In this context, it appears that beyond an exclusive focus on *PCE*, the chemistry of OPV should also take into consideration the development of active materials that combine decent efficiency with high yield, clean and scalable synthesis.

Small molecules have been used for a long time in vacuum-deposited OPV cells^17^,^18^,^19^,^20^, and recent work has shown that single-junction cells based on tailored donors of low molecular weight can reach power conversion efficiencies in the range of 6.0–7.0%^21^,^22^. It has been shown already that interesting photovoltaic performances can be obtained with small donor-acceptor molecules based on the triphenylamine (TPA) donor block^5^,^7^,^8^,^9^,^22^,^23^,^24^,^25^,^26^. Some of these compounds that combine low molecular weight, simple structure and good overall synthetic yield present the advantage to be processable from solutions or by thermal evaporation under vacuum^22^,^23^,^24^,^25^. Thus, when used in combination with C_60 _as acceptor a small structure such as **1** (Scheme 1) leads to a *PCE* of 2.50% in a simple bi-layer cell^23^ and 4.00% in a co-evaporated active layer^26^. Based on these promising results, compound **1** has been selected as a working platform for further structural manipulations with the double objective of improving the performances of the donor structure and progressing in our understanding of structure-properties relationships in this class of molecules. In this context, we have shown already that the rigidification of part of the structure by ethylene^24^ or *o*-phenylene^23^ covalent bridge can significantly improve the relevant photovoltaic parameters. As a further step, we report here on a series of small donor-acceptor molecules in which the phenyl groups of the TPA block of compound **1** are replaced by one or two naphtyl groups namely α-napthyl (**2a**)^27^, β-naphtyl (**2b**), α,β-dinaphtyl (**3ab**), and β,β-dinaphtyl (**4bb**) (Figure 1).

The synthesis and characterization of the electronic properties of the molecules are described and the effects of the number and mode of linkage of the naphtyl groups on the performances of simple bi-layer solar cells with C_60_ as acceptor material are discussed.

## Results and Discussion

**Figure 2:**
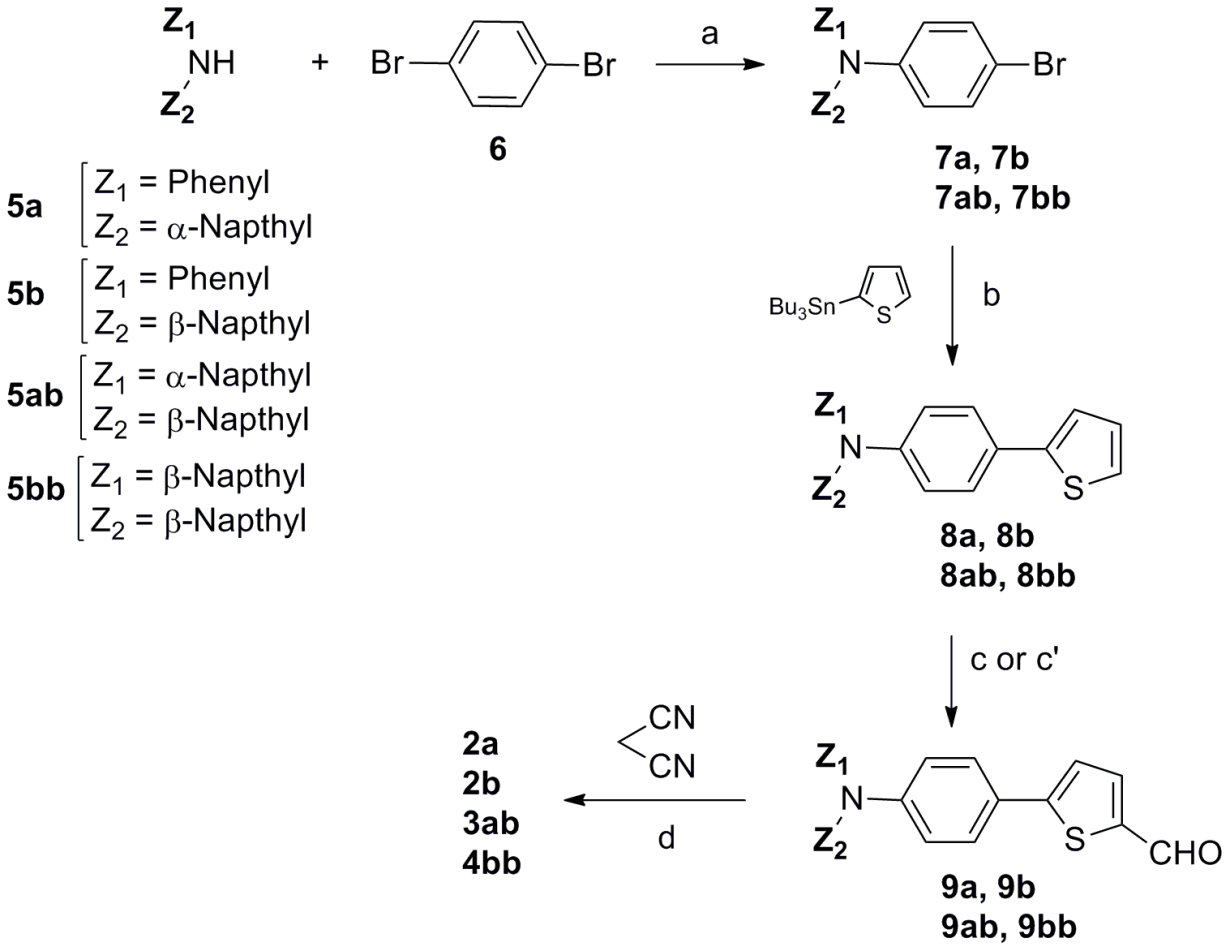
Synthesis of the target compounds. a) dpppPdCl_2_, *t*-BuONa, toluene, rflx 3 h; b) Pd(PPh_3_)_4_, toluene, rflx 16 h; c)** 8a**: POCl_3_, DMF, rflx 15 h; c') **8b**, **8ab**, **8bb**, *n*-BuLi, DMF/THF, −78°C, 3 h; d) Et_3_N, CHCl_3_, rflx 7 h.

The synthesis of the target compounds is described in Figure 2 (see SI). The brominated triarylamines (**7**) have been synthesized in 45 to 80% yield by coupling diarylamines **5** with *p*-dibromobenzene (**6**) in the presence a palladium catalyst. The reaction was optimized by varying the ligand of the catalyst and the temperature and duration of the reaction. A Stille coupling of bromo-compounds **7** with 2-(tributylstannyl)thiophene gives the 2-thienyl-derivatized compounds **8** in 70 to 95% yield. Compound **8a** is then subjected to a Vilsmeir-Haack formylation to give carboxaldehyde **9a** in 76% yield. For compounds **8b**, **8ab** and **8bb**, Vilsmeir formylation at the 2-position of thiophene is not selective and the reaction leads to a mixture of compounds due to concomitant formylation at the naphtyl groups. Therefore, formylation of these compounds has been achieved by lithiation with *n*-butyllithium followed by addition of DMF at low temperature. The four target compounds where then obtained in quantitative yield by Knoevenagel condensation of aldehydes **9** with malonodinitrile in the presence of triethylamine. The identity and purity of all new compounds has been confirmed by usual analytical methods (see SI).

**Figure 3:**
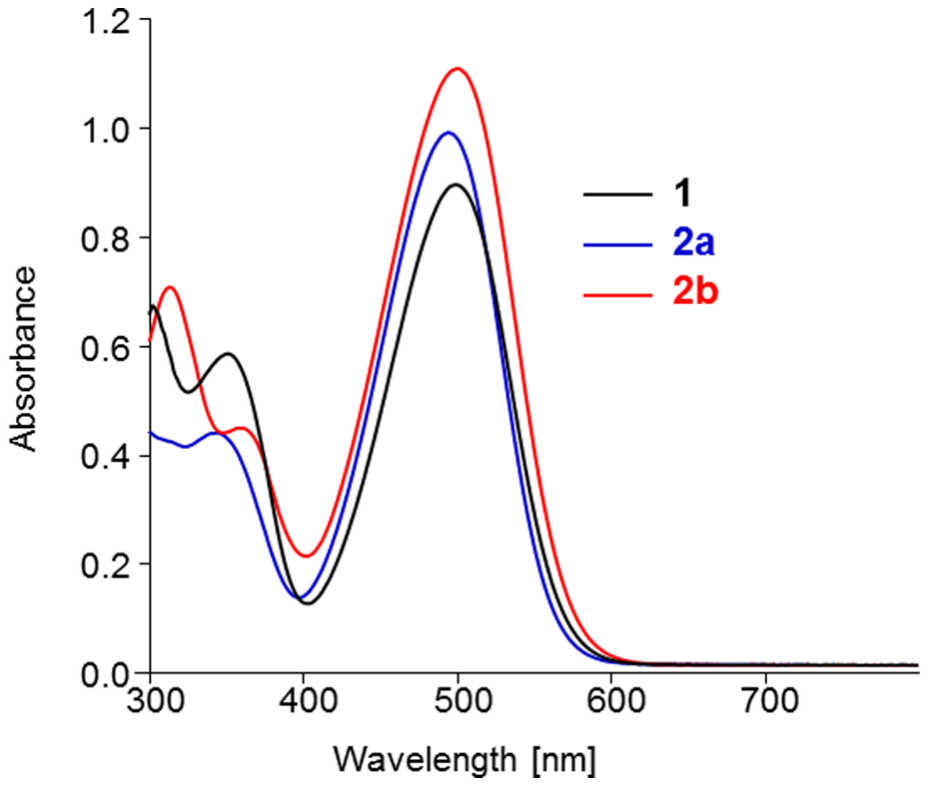
UV-Vis absorption spectra of compounds 1, 2a and 2b in CH_2_Cl_2_. The spectra of **3ab** and **4bb** have been omitted for clarity (see SI).

The UV-Vis absorption spectrum of the naphtyl substituted compounds exhibits a first band in the 300–400 nm region attributed to a π-π* transition followed by a more intense band with an absorption maximum (*λ*_max_) around 500 nm assigned to an internal charge transfer transition (Figure 3). Comparison of the optical data of compound **2–4** to those of the reference compound **1** shows that the introduction of naphtyl groups in the structure has little influence on the absorption maximum except for **4bb** for which a 10 nm bathochromic shift is observed (Table 1 and SI). On the other hand, the naphtyl groups induce a moderate increase of the molecular absorption coefficient (*ε*) in particular for compound **2b** for which the steric demand of the naphtyl group is expected to be minimal. As expected, the absorption spectra of thin films cast on glass from chloroform solutions present a slight red shift of *λ*_max_ and broadening of the absorption band while the low energy absorption edge lead to estimated band gaps of ~2.00 eV similar to that of compound **1**.

**Figure 4:**
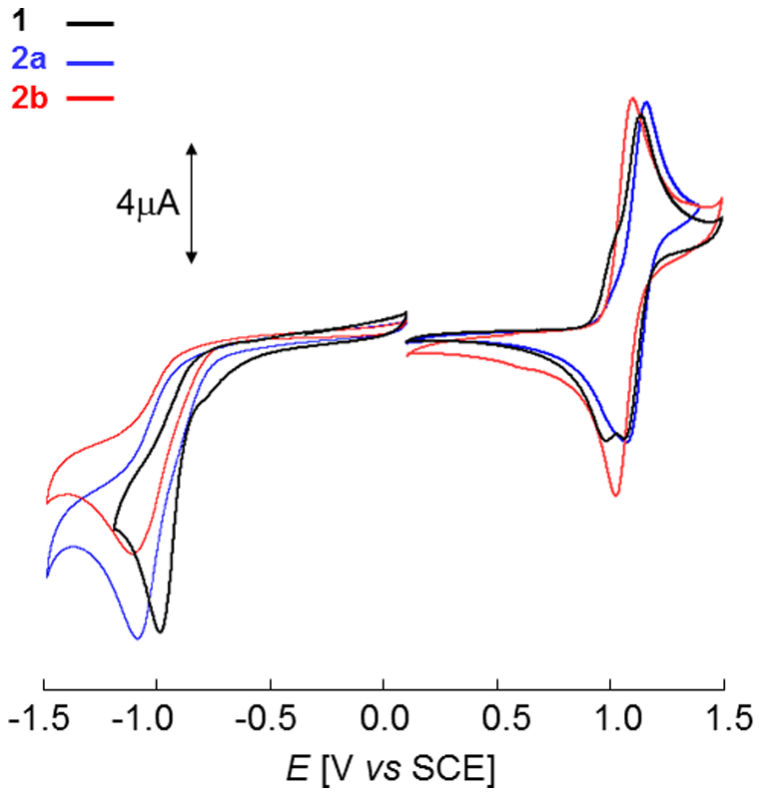
Cyclic voltammograms of compounds 1, 2a and 2b in 0.10 M Bu_4_NPF_6_/CH_2_Cl_2_, platinum electrodes, scan rate 100 mV cm^−1^.

Cyclic voltammetry (CV) was performed in dichloromethane in the presence of Bu_4_NPF_6_ as supporting electrolyte. All compounds present very similar CVs that exhibit a reversible one-electron oxidation process with an anodic peak potential (*E_pa_*) in the 0.90–1.05 V *vs* SCE range corresponding to the formation of the cation-radical (Figure 4) and an irreversible reduction process with a cathodic peak potential (*E_pc_*) in the −1.10 to −1.30 V region (Table 1 and SI). Replacement of a phenyl ring of compound **1** by a naphtyl group leads to different effects depending on the mode of linkage. Thus, for compound **2a** a 20 mV positive shift of *E_pa_* is observed. This effect can be assigned to the larger steric demand of the α-napthyl group *vs* the phenyl one which results in more twisted structure and hence lower donor strength of the aromatic block. In contrast, for **2b** a 40 mV negative shift of *E_pa_* occurs due to the increased donor effect of the β-napthyl group which allows a more coplanar geometry of the molecule.

The introduction of a second naphtyl group produces a further small negative shift of *E_pa_* to 0.93 and 0.91 V for **3ab** and **4 bb** respectively. Again the lower oxidation potential of the β,β'-disubstituted compound **4bb** compared to the α,β' **3ab** can be attributed to a lesser steric hindrance. Replacement of one phenyl ring by a naphtyl group produces a *ca* 100 mV negative shift of *E_pc_* while a second naphtyl group produces a further shift of 100–140 mV, these effects are consistent with the stronger donor effect of the naphtyl group compared to the phenyl one (Table 1 and SI).

These optical and electrochemical results show that the introduction of naphtyl groups in the structure of compound **1** has only a small impact on the absorption spectrum of the molecule and hence on its light-harvesting properties. Furthermore, the naphtyl groups exert only a limited influence on the energy level of the frontier orbitals. Thus, a single naphtyl group leads to a ~0.10 eV increase of the HOMO and LUMO level while introduction of two naphtyl groups raises these levels by 0.10–0.20 eV (Table 1).

A preliminary evaluation of the new compounds as donor material for OPV cells has been carried out on bi-layer planar heterojunction of 28 mm^2^ active area cells fabricated by spin-casting *ca* 20 nm thick films of donor from chloroform solutions containing 5 mg/mL of compound on ITO substrates pre-coated with 40 nm thick films PEDOT:PSS. The substrates were then introduced in a vacuum chamber. A 30 nm thick layer of C_60_ was deposited by thermal evaporation under a pressure of 2 × 10^−6^ mbar and the devices were completed by deposition of a 100 nm layer of aluminium. Each batch typically involves six cells. After fabrication the cells were subjected to a ten minutes thermal treatment at 120°C. Although the solubility of the compounds was compatible with the fabrication of solution-processed BHJ cells, no attempt in that direction was made at this stage of the research. Due to a larger dispersion of the results, solution-processed BHJ cells generally require longer optimization implying the fabrication of a larger number of devices than simple PHJ cells. Furthermore, since the purpose of this work is to analyse structure-properties relationships, simple two-layer devices appear as a more convenient tool as they give more reproducible and more reliable results despite an efficiency inferior to that of BHJ cells.

**Figure 5:**
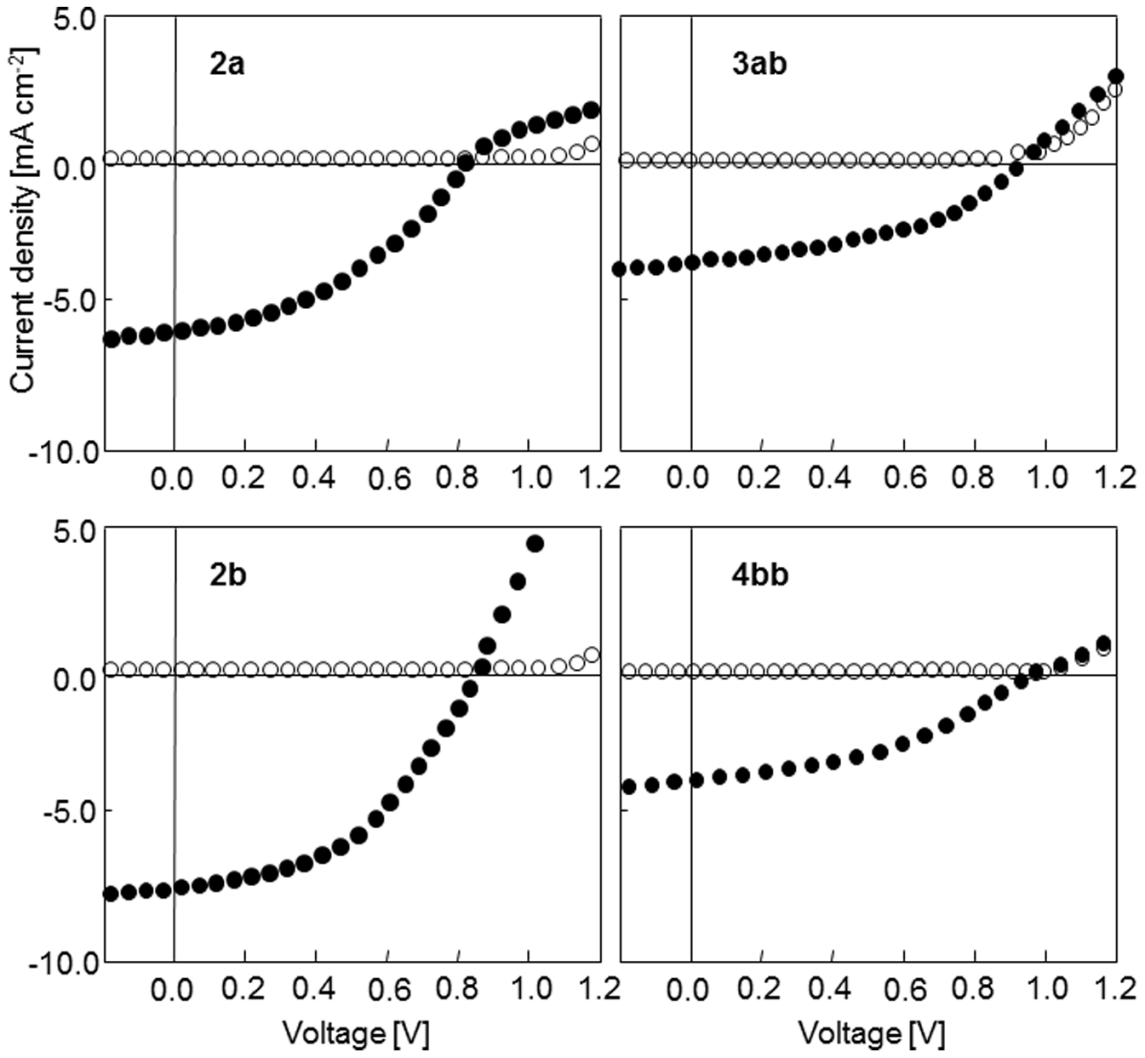
Current density *vs* voltage curves of bi-layer cells ITO/PEDOT:PSS/donor/C_60_/Al. In the dark (open circles) and under AM 1.5 simulated solar light (90 mW cm^−2^) (black circles).

The photovoltaic characteristics of the bi-layer cells based on the four donors containing napthyl groups are shown in Figure 5 and the corresponding data are listed in Table 2 using compound **1** as reference. A first examination of the current density *vs* voltage curves of Figure 3 clearly shows that the introduction of naphtyl groups in the donor structure exerts a considerable effect on the performances of the cells. Thus, replacement of two phenyl groups by two naphtyl ones leads to a decrease of *PCE* from 2.50% for **1** to ~1.70 and 1.40% for **3ab** and **4bb** respectively. This phenomenon results essentially from the decrease of *J_sc_* to values inferior to 4.0 mA cm^−2^. Surprisingly, the devices based on the di-naphtyl compounds **3ab** and **4bb** show slightly higher *V_o_*_c_ values than compound **1** although the opposite effect could be anticipated in view of the higher HOMO level of **3ab** and **4bb** compared to the singly substituted compounds^28^. Such unexpected structural effects on *V_o_*_c_ have already been observed and attributed to changes in the interfacial dipole at the D/A heterojunction^29^. Anyway further work is needed to clarify this point. Except for **2b**, the J *vs* V curves present a more or less pronounced *S* shape. This phenomenon can be attributed to imbalanced mobility of holes and electrons^30^ and/or to problems of charge-transfer at the active material/electrode interface. Based on the relatively small differences among the HOMO and LUMO levels of the various donors, the former explanation appears more plausible. Furthermore, this interpretation is consistent with the observed differences in hole-mobilities (see below).

The results for compound **2a** show that replacement of one phenyl group by an α-naphtyl one leads to a small decrease of *V_oc_* and fill factor resulting in a decrease of *PCE* from *ca* 2.50 to 2.20%. However, when the naphtyl group is connected to the nitrogen atom by its β-position in **2b**, a marked increase of *PCE* is observed due in particular to an increase of *J_sc_* up to 7.80 mA cm^−2^ and to an improvement of the filling factor (*FF*). The best device gave a *PCE* of ~3.40% with an average value of 3.10% (see SI). Based on the large increase of *PCE* previously observed upon optimization of cells based on compound **1**^26^ the results obtained with **2b** suggest that there is still much room for improvement of the performances of cells.

**Figure 6:**
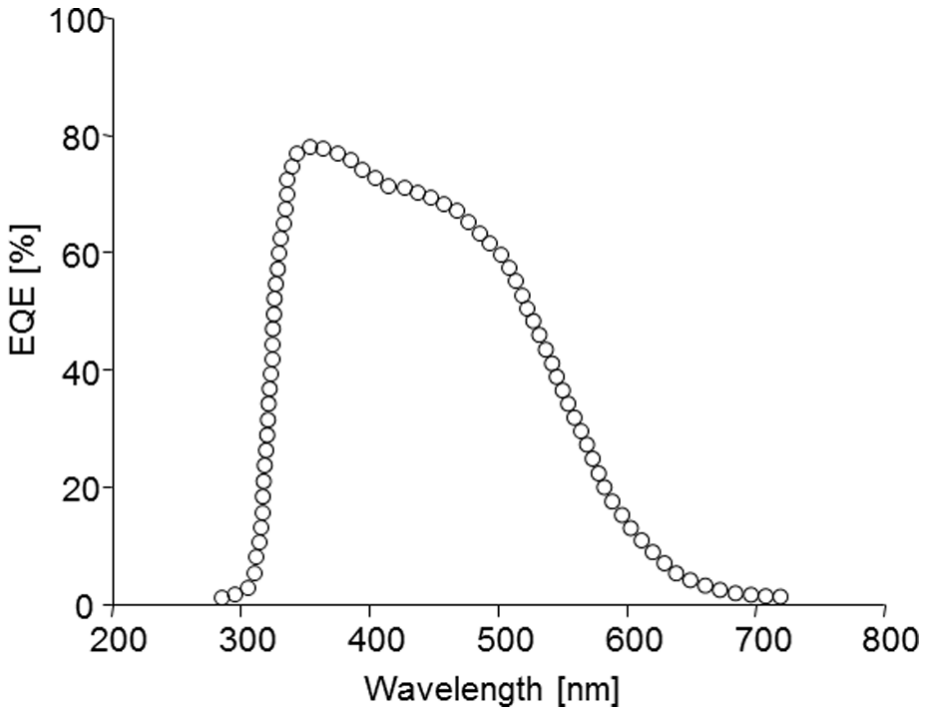
External quantum efficiency of the cell ITO/PEDOT:PSS/2b/C_60_/Al under monochromatic irradiation.

Figure 6 shows the external quantum efficiency (*EQE*) action spectrum of a device based on **2b** under monochromatic irradiation. The first maximum around 360 nm can be attributed to the contribution of C_60_. This first band is followed by a broad shoulder in the 400 to 600 nm region with a maximum of ~70% in agreement with the absorption spectrum of the donor. These results show that the β-naphtyl D-A molecule can lead to interesting photon/electron conversion efficiency, however they also underline the need of further structural modifications in order to extend the light-harvesting properties of the molecules towards longer wavelengths.

Although these results clearly demonstrate that the introduction of naphtyl groups in the structure of compound **1** exerts a marked influence on the photovoltaic performances of the donor, the origin of these effects is not evident. As shown by optical and electrochemical data, the napthyl groups have only limited effects on the light-harvesting properties and energy levels of the system. This indicates that the origin of the effects of the napthyl groups on the performances of the photovoltaic cells is probably related to changes in intermolecular interactions. In a previous work we have shown that the introduction of a 5-hexyl-2,2'-bithienyl hole-transporting group in the meso-position of a BODIPY donor leads to a two-fold increase of the efficiency of the resulting solar cell and that this effect was due exclusively to an increase of hole-mobility without any effect on electronic properties of the molecule^31^.

In order to test this hypothesis in the present case. The hole-mobility of the three representative compounds **1**, **2a** and **2b** has been measured by the space-charge limited current method (SCLC) on "hole-only" devices of structure: ITO/PEDOT PSS/donor/Au. Films of the donors of 200–300 nm thickness have been deposited by spin-casting from chloroform solutions (22 mg/mL) (see SI). The results show that the replacement of a phenyl group of compound **1** by an α-naphtyl group leads to a two-fold decrease of hole mobility (*μ*_H_) from 1.0 × 10^−5^ cm^2^ V^−1^ s^−1^ for **1** to 4.2 × 10^−6^ cm^2^ V^−1^ s^−1^ for **2a** (Table 2). In contrast, replacement of the phenyl by a β-naphtyl leads to an increase of *μ*_H_ by a factor of five (*μ*_H_ = 5.5 × 10^−5^ cm^2^ V^−1^ s^−1^) which results in a difference of more than one order of magnitude between the hole mobility of the α- and β- mono-substituted compounds **2a** and **2b**. This large difference which clearly confirms the significant role of the mode of grafting of the naphtyl group can be attributed to a molecular packing more propitious to efficient charge transport in the case of **2b** due to the already discussed lesser steric hindrance associated with the β-linkage. Finally comparison of these results with the photovoltaic data in Table 2 confirms, in agreement with previous conclusions^31^, that the structural optimization of the hole-mobility represents an interesting tool for the improvement of the photovoltaic performances of molecular donors.

## Conclusion

A series of donor-acceptor systems based on triarylamine donor blocks in which one or two phenyl rings are replaced by naphtyl groups has been synthesized in good overall yields. Although the introduction of naphtyl groups in the structure has little influence on the light-harvesting properties and energy levels of the molecule, the results obtained on bi-layer OPV cells reveal a large diversity of situations. The introduction of two naphtyl groups through α,β' or β,β,' linkages induces a net decrease of conversion efficiency. When linked via an α-position, a naphtyl group produces a small decrease of *PCE* while in contrast, when attached at a β-position this group produces a significant increase of *PCE* due in particular to a large increase of *J_sc_* correlated to a parallel increase of hole mobility. These opposite effects are attributed to the consequences of the different intramolecular steric effects associated with the mode of linkage of the naphtyl group on the intermolecular interactions and molecular packing. Based on the strong current interest in TPA-based systems for the design of donor materials for OPV, of non-metal sensitizers for dye-sensitized solar cells^32^, or of hole-transporting materials for perovskite solar cells^33^ these results provide a strong incitement to further investigations of structure-properties relationships in this class of D-A systems. Work in this direction is now underway and will be reported in future publications.

## Author Contributions

S.M. synthesized and characterized the materials. D.D. performed device fabrication and characterization, J.R. supervised the project and prepared the manuscript. J.-A.L. and P.B. analysed the data, discussed the results and contributed to revisions.

## Supplementary Material

Supplementary Information Structure-properties relationships in triarylamine-based donor-acceptor molecules containing naphtyl groups as donor material for organic solar cells

## Figures and Tables

**Figure 1:**
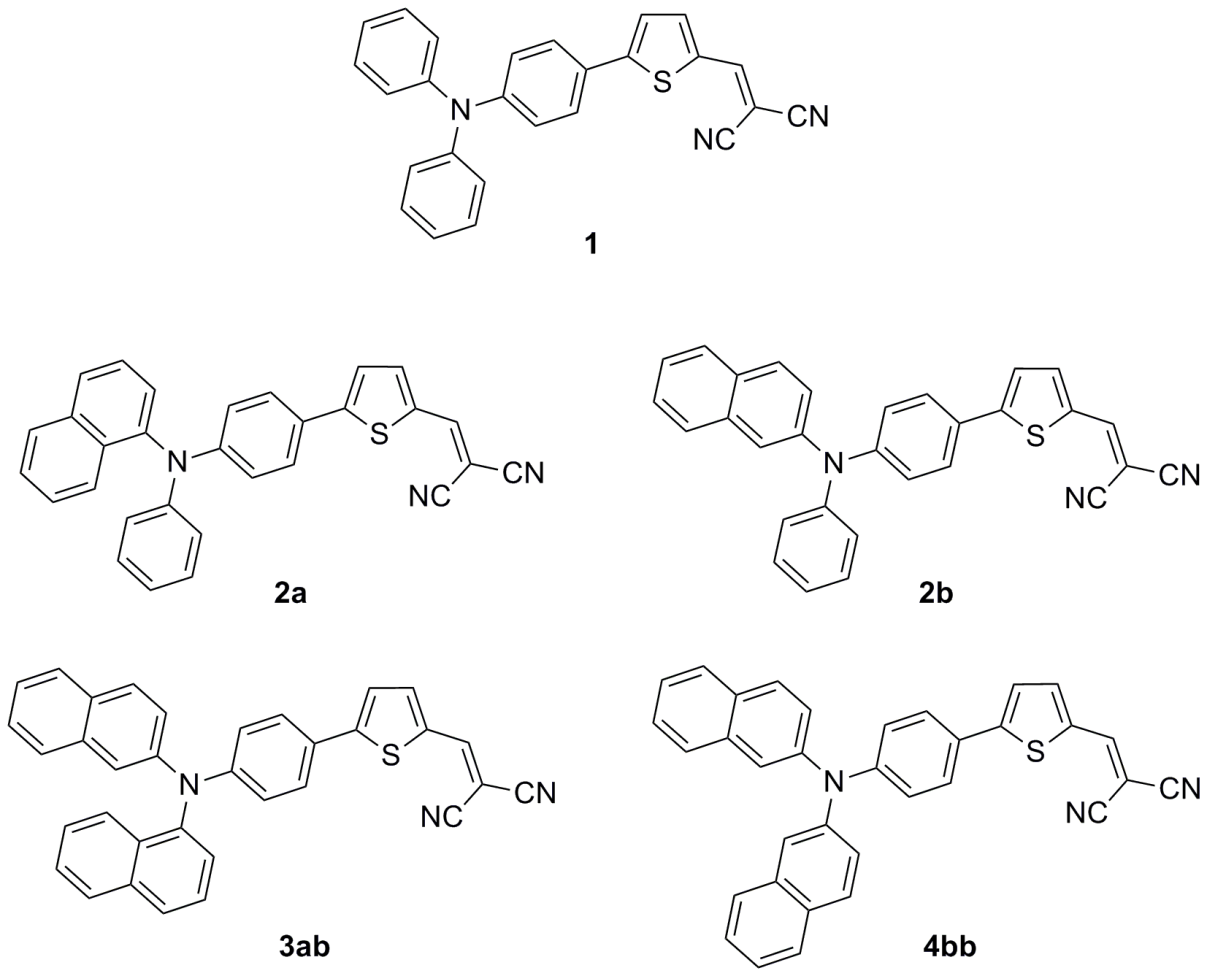
Chemical structures of the target compounds.

**Table 1:** UV-Vis absorption and cyclic voltammetry data for the D-A compounds

Compd	*λ*_max_^a^ [nm]	*λ*_max_^b^ [nm]	*ε* [M^−1^ cm^−1^]	*E_pa_*^c^ [V *vs* SCE]	*E_pc_* [V *vs* SCE]	HOMO^d^ [eV]	LUMO [eV]	*Δ*E [eV]
**1**^e^	500	523	27000	1.04	−1.10	−6.0	−3.8	2.2
**2a**	498	510	32500	1.06	−1.19	−6.0	−3.8	2.2
**2b**	500	518	36300	1.00	−1.21	−5.9	−3.7	2.2
**3ab**	494	503	27000	0.93	−1.34	−5.7	−3.4	2.3
**4bb**	510	513	31000	0.91	−1.29	−5.7	−3.5	2.2

^a^in CH_2_Cl_2_;

^b^on films;

^c^in the conditions of Figure 2;

^d^using an offset of −4.99 eV *vs* vacuum level for SCE.

^e^from Ref. 23.

**Table 2:** Photovoltaic characteristics of bi-layer solar cells donor/C_60_ under AM 1.5 simulated solar illumination with a power intensity of 90 mW cm^−2^. ^a^ from Ref. 23. Data in italics are the average values of six cells (8 for **2b**) and data in bold are the best values in each series

Donor	*J_sc_* (mA cm^−2^)	*V_oc_* (V)	*FF*	*PCE* (%)	*μ_H_* (cm^2^ V^−1^ s^−1^)
**1**	5.80	0.92	0.42	2.50^a^	1.0 × 10^−5^
**2a**	*5.90*	*0.80*	*0.40*	*2.10*	
**2a**	**6.20**	**0.80**	**0.40**	**2.20**	0.42 × 10^−5^
**2b**	*7.50*	*0.83*	*0.45*	*3.11*	
**2b**	**7.80**	**0.83**	**0.47**	**3.38**	5.5 × 10^−5^
**3ab**	*3.75*	*0.94*	*0.39*	*1.53*	
**3ab**	**4.00**	**0.96**	**0.39**	**1.66**	
**4bb**	*3.10*	*0.95*	*0.40*	*1.31*	
**4bb**	**3.30**	**0.92**	**0.40**	**1.35**	

## References

[b1] Brabec C., Scherf U. & Dyakonov V. Organic Photovoltaics. John Wiley & Sons, 2014.

[b2] Günes S., Neugebauer H. & Sariciftci N. S. Conjugated polymer-based organic solar cells. Chem. Rev. 107, 1324–1338 (2007). 17428026 10.1021/cr050149z

[b3] Chen Y.-J., Yang S.-H., Hsu C.-S. Synthesis of conjugated polymers for organic solar cell applications. Chem. Rev. 109, 5868–5923 (2009). 19785455 10.1021/cr900182s

[b4] Liang Y. & Yu L. A New Class of Semiconducting Polymers for Bulk Heterojunction Solar Cells with Exceptionally High Performance. Accounts Chem. Res. 43, 1227–1236 (2010). 10.1021/ar1000296 20853907

[b5] Roncali J. Molecular Bulk Heterojunctions: An Emerging Approach to Organic Solar Cells. Accounts. Chem. Res. 42, 1719–1730 (2009). 10.1021/ar900041b 19580313

[b6] Würthner F. & Meerholz K. Systems Chemistry Approach in Organic Photovoltaics. Chem-Eur. J. 16, 9366–9373 (2010). 20645353 10.1002/chem.201001153

[b7] Walker B., Kim C. & Nguyen T. Q. Small Molecule Solution-Processed Bulk Heterojunction Solar Cells. Chem. Mater. 23, 470–482 (2011).

[b8] Lin Y., Li Y. & Zhan X. Small molecule semiconductors for high-efficiency organic photovoltaics. Chem. Soc. Rev. 41, 4245–4272 (2012). 22453295 10.1039/c2cs15313k

[b9] Roncali J., Leriche P. & Blanchard P. Molecular materials for organic photovoltaics: small is beautiful. Adv. Mater. 26, 3821–3838. (2014) 24687246 10.1002/adma.201305999

[b10] Roncali J. *et al.* Molecular and Supramolecular Engineering of pi-Conjugated Systems for Photovoltaic Conversion. Thin Solid Films 511–512, 567–575 (2006).

[b11] Chen Y., Wan X. & Long G. High Performance Photovoltaic Applications Using Solution-Processed Small Molecules. Accounts Chem. Res. 46, 2645–2655 (2013). 10.1021/ar400088c 23902284

[b12] Coughlin J. E., Henson Z. B., Welch G. C. & Bazan G. Design and Synthesis of Molecular Donors for Solution-Processed High-Efficiency Organic Solar Cells. Accounts Chem. Res. 47, (2014) 257–270. 10.1021/ar400136b 23984626

[b13] Kan B. *et al.* Solution-Processed Organic Solar Cells Based on Dialkylthiol-Substituted Benzodithiophene Unit with Efficiency near 10%. J. Am. Chem. Soc doi.org/10.1021/ja509703k (2014) 10.1021/ja509703k 25337798

[b14] Burke D. J. & Lipomi D. J. Green chemistry for organic solar cells. Energy & Environ. Sci. 6, 2053–2066 (2013).

[b15] Osedach T. P., Andrew T. L. & Bulovic V. Effect of synthetic accessibility on the commercial viability of organic photovoltaics. Energy & Environ. Sci. 6, 711–718 (2013).

[b16] Marzano G. *et al.* Organometallic Approaches to Conjugated Polymers for Plastic Solar Cells: From Laboratory Synthesis to Industrial Production. Eur. J. Org. Chem. 30, 6583–6614 (2014).

[b17] Tang C. W. A two-layer organic solar cell. Appl. Phys. Lett. 48, 183–185 (1986).

[b18] Wöhrle D. &. Meissner D. Organic solar cells. Adv. Mater. 3, 129–139 (1991).

[b19] Peumans P., Yakimov A. & Forrest S. R. Small molecular weight organic thin film photodetectors and solar cells. J. Appl. Phys. 93, 3693–3723 (2003).

[b20] de Bettignies R. *et al.* Planarized Star-shaped Oligothiophenes as Organic Semi-Conductors for Efficient Heterojunction Solar Cells. Adv. Mater. 15, 1939–1943 (2003).

[b21] Steinmann V. *et al.* Simple, Highly Efficient Vacuum-Processed Bulk Heterojunction Solar Cells Based on Merocyanine Dyes. Adv. Energy Mater. 1, 888–894 (2011).

[b22] Chen Y. H. *et al.* Vacuum-Deposited Small-Molecule Organic Solar Cells with High Power Conversion Efficiencies by Judicious Molecular Design and Device Optimization. J. Am. Chem. Soc. 134, 13616–13623 (2012). 22831172 10.1021/ja301872s

[b23] Leliège A., Le Régent C. H. Allain M., Blanchard P. & Roncali J. Structural modulation of internal charge transfer in small molecular donors for organic solar cells. Chem. Commun. 48, 8907–8909 (2012). 10.1039/c2cc33921h 22842881

[b24] Demeter D. *et al.* Tuning of the photovoltaic parameters of molecular donors by covalent bridging. Adv. Funct. Mater. 23, 4854–4861 (2013).

[b25] Demeter D., Mohamed S., Diac A., Grosu I. & Roncali J. Small molecular donors for organic solar cells obtained by simple and clean chemistry. ChemSusChem 7, 1046–1051 (2014). 24591362 10.1002/cssc.201301339

[b26] Choi J. W. *et al.* Exploiting the potential of 2-((5-(4 (diphenylamino)phenyl)thiophen-2-yl)methylene)malononitrile as an efficient donor molecule in vacuum-processed bulk-heterojunction organic solar cells. RSC Advances 4, 5236–5242 (2014).

[b27] Jung M.-H., Song K. H., Ko K. C., Lee J. Y. & Lee, H. Non volatile memory organic field effect transistor induced by the steric hindrance effects of organic molecules. J. Mater. Chem. 20, 8016–8020 (2010).

[b28] Brabec C. J. *et al.* Origin of the open-circuit voltage of plastic solar cells. Adv. Funct. Mater. 11, 374–380 (2001).

[b29] Demeter D. *et al.* Manipulation of the Open-Circuit Voltage of Organic Solar Cells by Desymmetrization of the Structure of Acceptor–Donor–Acceptor Molecules. Adv. Funct. Mater. 21, 4379–4387 (2011).

[b30] Tress W. *et al.* Imbalanced mobilities causing S-shaped J/V curves in planar heterojunction organic solar cells. Appl. Phys. Lett. 98, 063301 (2011).

[b31] Rousseau T. *et al.* A Tailored Hybrid BODIPY-Oligothiophene donor for molecular bulk heterojunction solar cells with improved performances. Chem. Commun. 46, 5082–5084 (2010). 10.1039/c0cc01144d 20559594

[b32] Yen Y.-S., Chou H.-H., Chen Y.-C., Hsu C. Y. & Lin J. T. Recent developments in molecule-based organic materials for dye-sensitized solar cells. J. Mater. Chem. 22, 8734–8747 (2012).

[b33] Choi H. *et al.* Efficient Perovskite Solar Cells with 13.63 % Efficiency Based on Planar Triphenylamine Hole Conductors. Chem. Eur. J. 20, 10894–10899 (2014). 25100664 10.1002/chem.201403807

