# Supplementary material for: Structure-properties relationships in triarylamine-based donor-acceptor molecules containing naphtyl groups as donor material for organic solar cells
Source: Sci Rep. 2015 Mar 12;5:9031. doi: 10.1038/srep09031 (PMC4356976; doi:10.1038/srep09031)
Supplement: Supplementary Information — Structure-properties relationships in triarylamine-based donor-acceptor molecules containing naphtyl groups as donor material for organic solar cells [file srep09031-s1.doc]

**Supplementary information**

**Structure-properties relationships in triarylamine-based donor-acceptor molecules containing naphtyl groups as donor material for organic solar cells**

Salma Mohamed,^†^ Dora Demeter,^†^ Jean-Alex Laffitte,^‡^ Philippe Blanchard^†^ and Jean Roncali^†^*

**General**

NMR spectra were recorded with a Bruker Avance III 300 (^1^H, 300 MHz and ^13^C, 75 MHz) or Bruker Avance DRX 500 (^1^H, 500 MHz and ^13^C, 125 MHz). Chemical shifts are given in ppm relative to TMS. IR spectra were recorded on a Bruker spectrometer Vertex 70 and UV-Vis spectra with a Perkin Elmer 950 spectrometer. Melting points are uncorrected. Matrix Assisted Laser Desorption/Ionization was performed on MALDI-TOF MS BIFLEX III Bruker Daltonics spectrometer using dithranol as matrix.

Cyclic voltammetry was performed in 0.10 M Bu_4_NPF_6_/CH_2_Cl_2_ (HPLC grade). Solutions were degassed by nitrogen bubbling prior to each experiment. Experiments were carried out in a one-compartment cell equipped with platinum electrodes and a saturated calomel reference electrode (SCE) using a Biologic SP-150 potentiostat with positive feedback compensation. Column chromatography purifications were carried out on Acros silica gel Si 60 (35-70 mm). DSC and TGA were performed with TA Instruments.

**Synthesis**

*N-(4-bromophenyl)-N-phenylnaphthalen-1-amine* (**7a**). Sodium terbutoxide (1.75 g, 18.2 mmol), 1.4-dibromobenzene (**6**) (2.69 g, 11.4 mmol) and *N*-phenylnaphthalen-1-amine (**5a**) (1 g, 4.56 mmol) were dissolved in 40 ml of dry toluene. The mixture was degassed for 10 min with argon. Pd(dppf)Cl_2_ (100 mg, 3 mol %) was introduced and the mixture was heated at 120 °C for 4 h under argon. After filtration and solvent evaporation, the product was chromatographed on silica gel (eluent cyclohexane/CH_2_Cl_2_ 8.5:1.5). Yield 1.30 g (77%). HRMS=373.0477. ^1^H NMR (CDCl_3_): δ (ppm) = 7.90-7.93 (d, 2H), 7.79-7.81 (d, 2H), 7.47-7.50 (t, 2H), 7.37-7.40 (t, 1H), 7.32-7.33 (d, 1H), 7.27-7.29 (d, 2H), 7.21-7.24 (t, 2H), 7.06-7.08 (d, 2H), 6.97-7 (t, 1H).

*N-(4-bromophenyl)-N-phenylnaphthalen-2-amine* (**7b**). This compound was prepared using the same procedure from *N*-phenylnaphthalen-2-amine (1 g, 4.56 mmol). Yield 1.35 g (79%). HRMS=373.0477. ^1^H NMR (CDCl_3_): δ (ppm) = 7.90-7.93 (d, 2H), 7.79-7.81 (d, 2H), 7.47-7.50 (t, 2H), 7.37-7.40 (t, 1H), 7.32-7.33 (d, 1H), 7.27-7.29 (d, 2H), 7.21-7.24 (t, 2H), 7.06-7.08 (d, 2H), 6.97-7 (t, 1H).

*N-(4-bromophenyl)-N-(naphthalen-2-yl) naphthalen-1-amine* (**7ab**). This compound was prepared using the same procedure from *N*-(naphthalen-2-yl)naphthalen-1-amine (**5ab**), (1.0 g, 3.7 mmol), using cyclohexane as eluent for chromatography. Yield 0.72 g, 46%. MS-MALDI-TOF m/z = 423.1**.** ^1^H NMR (CDCl_3_): δ (ppm) = 7.95-8.01 (dd, 2H), 7.85-7.88 (d, 1H), 7.73-7.80 (m, 2H), 7.50-7.59 (m, 3H), 7.34-7.44 (m, 8H), 6.97-7 (d, 2H).

*N-(4-bromophenyl)-N-(naphthalen-1-yl) naphthalen-1-amine* (**7bb**). This compound was prepared using the same procedure from *N-(4-bromophenyl)-N-phenylnaphthalen-1-amine* (**7a**). Yield 0.86 g (54%). SM-MALDI m/z: 425.1; ^1^H NMR (CDCl_3_): δ (ppm) = 7.79-7.37 (m, 4H), 7.61-7.58 (m, 2H), 7.46-7.35 (m, 8H), 7.31 (dd, 2H), 7.06 (d, 2H); ^13^C NMR (CDCl_3_): δ (ppm) = 146.8, 144.9, 134.3, 132.3, 130.3, 129.1, 127.6, 127.0, 126.4, 125.5, 124.8, 124.4, 120.9, 115.3.

*N-phenyl-N-(4-(thiophen-2-yl)phenyl)naphthalen-1-amine* (**8a**). 920 mg (2.45 mmol) of (**7a**) and 2-tributylstannylthiophene (1.18 ml, 3.72 mmol) were dissolved in 40 ml of dry toluene. The solution was degassed for ten minutes. Tetrakis(triphenylphosphine)palladium(0) (143.3 mg, 0.124 mmol) was added and the mixture was refluxed for 24 hours. The solvent was evaporated, the product was purified with column chromatography on silica gel (solvent cyclohexane/CH_2_Cl_2_ 8:2). Yield 68% (629 mg, 1.66 mmol).

*5-(4-(naphthalen-1-yl(phenyl)amino)phenyl)thiophene-2-carbaldehyde* (**9a**). 510 mg (1.35 mmol) of (**8a**) was dissolved in 40 ml of dichloroethane. Phosphoryl chloride (138.5 µl, 1.486 mmol) and N,N-dimethylformamide (143 µl, 1.85 mmol) were added. The mixture was refluxed for 15 hours. 10 equivalents of sodium acetate, 10 ml of distilled water and 20 ml of dichloromethane were added, the mixture was further agitated for 4 hours. The product was extracted with dichloromethane, the organic phases were washed with distilled water, dried over MgSO_4_, filtered and concentrated under vacuum. The product was purified with column chromatography (eluent CH_2_Cl_2_/cyclohexane 9:1). Yield 76% (416 mg, 1.026 mmol).

*N-phenyl-N-(4-(thiophen-2-yl)phenyl)naphthalen-2-amine* (**8b**). 920 mg (2.45 mmol) of (**7b**) and tributylstannylthiophene (1.18 ml, 3.72 mmol) were dissolved in 40 ml of dry toluene. The solution was degassed 10 min. Tetrakis(triphenylphosphine)palladium(0) (143.3 mg, 0.124 mmol) was added and the mixture was refluxed for 24 h. The solvent was evaporated, the product was chromatographed on silica gel (solvent cyclohexane/CH_2_Cl_2_ 8:2). Yield 68% (629 mg, 1.66 mmol). HRMS= 377.1225. ^1^H NMR (CDCl_3_) : δ (ppm) = 7.78-7.72 (m, 2H), 7.62-7.59 (m, 1H), 7.51-7.48 (d, 2H), 7.46 (d, 1H), 7.40-7.35 (m, 2H), 7.32-7.28 (m, 3H), 7.24-7.25 (m, 2H), 7.18-7.15 (m, 2H), 7.13-7.10 (d, 2H), 7.07-7.04 (m, 2H).

*5-(4-(naphthalen-2-yl(phenyl)amino)phenyl)thiophene-2-carbaldehyde* (**9b**). In a Schlenk filled with argon, 200 mg (0.53 mmol, 1eq. ) of (3b) was dissolved in 10 ml of dry THF and cooled to -78°C, *n*-BuLi (224 µl, 0.6 mmol, 1.2 eq.) was added dropwise and the mixture was stirred for 30 minutes . A white precipitate was formed. DMF (71 µl, 1.06 mmol, 2eq.) was added and the mixture was stirred overnight. The solvent was evaporated and the product was chromatographed on silica gel (eluent cyclohexane/CH_2_Cl_2_ 4:6). Yield 49% (yellow powder, 100 mg, 0.26 mmol). SM-MALDI m/z = 405.1; ^1^H NMR (CDCl_3_) : δ (ppm) = 9.7 (s, 1H), 7.67-7.65 (m, 1H), 7.56-7.54 (d, 2H), 7.48(d, 1H), 7.43-7.38 (m, 2H), 7.35-7.30 (m, 3H), 7.26-7.24 (m, 2H), 7.20-7.15 (m, 2H), 7.13-7.11 (d, 2H), 7.06-7.03 (m, 2H).

*N-(naphthalen-2-yl)-N-(4-(thiophen-2-yl)phenyl)naphthalen-1-amine* (**8ab**). 300 mg (0.7 mmol, 1 eq.) of (**7ab**) was dissolved in 30 ml of dry toluene, tributylstannylthiophene (338 µl, 1.05 mmol, 1.5 eq.) and tetrakis(triphenylphosphine)palladium (40 mg, 0.035 mmol, 0.05 eq.) were added and the mixture was refluxed 3 h. The solvent was evaporated and the product was chromatographed on silica gel yielding 84% (253 mg, 0.59 mmol) of a white powder. MS-MALDI m/z = 427.1; ^1^H NMR (CDCl_3_): δ (ppm) = 8.01-7.99 (d, 1H), 7.94-7.91 (d, 1H), 7.84-7.82 (d, 1H), 7.77-7.70 (m, 2H), 7.56-7.46(m, 4H), 7.41-7.34 (m, 6H), 7.22-7.21 (m, 2H), 7.09-7.04 (m, 3H).

*5-(4-(naphthalen-1-yl(naphthalen-2-yl)amino)phenyl)thiophene-2-carbaldehyde* (**9ab**). In a Schlenk filled with argon, 200 mg (0.46 mmol, 1eq. ) of (**8ab**) was dissolved in 10 ml of dry THF and cooled to -78°C, nBuLi (224 µl, 0.56 mmol, 1.2eq.) was added dropwise and the mixture was stirred for 30 minutes . A white precipitate was formed. DMF (71 µl, 0.92 mmol, 2 eq.) was added and the mixture was stirred for the night. The solvent was evaporated and the product was chromatographed on silica gel (eluent cyclohexane/CH_2_Cl_2_ 4:6). Yield 46% (yellow powder, 100 mg, 0.2 mmol). MS-MALDI m/z = 455.2. ^1^H NMR (CDCl_3_): δ (ppm) = 9.8 (s, 1H), 7.95-7.91 (dd, 2H), 7.85 -7.83 (d, 1H), 7.75-7.72 (m, 2H), 7.68-7.67 (d, 1H), 7.51-7.48 (m, 3H), 7.44-7.41 (m, 3H), 7.38-7.35(m, 4H), 7.28-7.27 (d, 1H), 7.03-7.00 (m, 2H).

*N-(naphthalen-1-yl)-N-(4-(thiophen-2-yl)phenyl)naphthalen-1-amine* (**8bb**)

400 mg (0.9 mmol, 1 eq.) of (**7bb**) was dissolved in 50 ml of dry toluene, tributylstannylthiophene (450 µl, 1.4 mmol, 1.5 eq.) and tetrakis(triphenylphosphine)palladium (54 mg, 0.047 mmol, 0.05 eq.) were added and the mixture was refluxed 4 h. The solvent was evaporated and the product was chromatographed on silica gel (eluent petroleum ether and after petroleum ether/ CH_2_Cl_2_ 7:3). Yield 97% (light yellow powder, 390 mg). MS-MALDI m/z: 427.1; ^1^H NMR (CDCl_3_): δ (ppm) = 7.80-7.75 (m, 3H), 7.63-7.60 (m, 3H), 7.54 (d, 2H), 7.51 (s, 1H), 7.42-7.31 (m, 8H), 7.19 (d, 2H), 7.10-7.06 (m, 2H); ^13^C NMR (CDCl_3_): δ (ppm) = 147.0, 145.0, 144.1, 134.3, 130.2, 129.0, 128.8, 128.0, 127.9, 127.5, 127.4, 127.0, 126.8, 126.3, 125.9, 124.7, 124.5, 124.2, 124.1, 123.0, 122.3, 120.8.

*5-(4-(naphthalen-1-yl(naphthalen-1-yl)amino)phenyl)thiophene-2-carbaldehyde* (**9bb**).

In a Schlenk filled with argon, 390 mg (0.93 mmol) of (**8bb**) was dissolved in 20 ml of dry THF and cooled to -78°C, *n*-BuLi (1.02 mmol, 1.1eq.) was added dropwise and the mixture was stirred for 30 min. DMF (144 µl, 1.86 mmol, 2eq.) was added and the mixture was stirred overnight. The solvent was evaporated and the product was chromatographed on silica gel (eluent petroleum ether/CH_2_Cl_2_ 4:6). Yield 46% (yellow powder, 191 mg, 0.4 mmol). MS-MALDI m/z = 455.2. ^1^H NMR (CDCl_3_): δ (ppm) = 9.8 (s, 1H), 7.82-7.78 (m, 4H), 7.81 (d, 1H), 7.65-7.62 (m, 2H), 7.58-7.55 (m, 4H), 7.44-7.41 (m, 4H), 7.37-7.32 (m, 3H), 7.19 (d, 2H); ^13^C NMR (CDCl_3_): δ (ppm) = 182.5, 154.3, 148.9, 144.5, 141.4, 137.6, 134.3, 130.5, 129.3, 127.6, 127.3, 127.0, 126.7, 126.4, 125.1, 124.7, 123.0, 121.8.

*2-((5-(4-(naphthalen-1-yl(phenyl)amino)phenyl)thiophen-2-yl)methylene)malono-nitrile* (**2a**). 280 mg (0.69 mmol) of (**9a**) and malononitrile (91.22 mg, 1.38 mmol) were dissolved in 20 ml of chloroform. 3 drops of triethylamine were added and the mixture was refluxed for 4 hours. The product was purified by column chromatography (eluent CH_2_Cl_2_/cyclohexane 9:1). Yield 62% (193 mg, 0.428 mmol).

*2-((5-(4-(naphthalen-2-yl(phenyl)amino)phenyl)thiophen-2-yl)methylene)malono-nitrile* (**2b**)**.** 100 mg (0.26 mmol) of (**9b**) and malononitrile (35 mg, 0.52 mmol) were dissolved in 5 ml of chloroform. 1 drop of triethylamine was added and the mixture was refluxed 2h. The product was chromatographed on silica gel (eluent CH_2_Cl_2_/cyclohexane 9:1). Yield 99% (110 mg, 0.25 mmol).MS-MALDI m/z = 453.1; ^1^H NMR (300MHz, CDCl_3_) : δ (ppm) = 7.96-7.85 (m, 3H), 7.75 (s, 1H), 7.68(d, 1H), 7.54-7.32 (m, 9H), 7.20-7.15 (m, 2H), 7.06-7.03 (m, 2H), 6.98 (d, 1H).

*2-((5-(4-(naphthalen-1-yl(naphthalen-2-yl)amino)phenyl)thiophen-2-yl)methylene)-malononitrile* (**3ab**). 100 mg (0.21 mmol, 1 eq.) of (**9ab**) and malononitrile (29 mg, 0.4 mmol, 2eq.) were dissolved in 5 ml of chloroform; the mixture was degassed for 10 minutes. One drop of triethylamine was added and the mixture was refluxed for 3 hours. The product was purified by chromatography on silica gel (eluent petroleum ether/CH_2_Cl_2_ 8:2) yielding 100 mg (91%) of a red powder. MS-MALDI m/z = 503.2. ^1^H NMR (CDCl_3_): δ (ppm) = 7.96-7.93 (dd, 2H), 7.89 (s, 1H), 7.86 -7.83 (d, 1H), 7.79-7.76 (m, 2H), 7.69-7.68 (d, 1H), 7.53-7.50 (m, 3H), 7.46-7.42 (m, 3H), 7.40-7.38(m, 4H), 7.21-7.20 (d, 1H), 7.05-7.02 (m, 2H).

*2-((5-(4-(naphthalen-2-yl(naphthalen-2-yl)amino)phenyl)thiophen-2-yl)methylene)-malononitrile* **(4bb).** 100 mg (0.2 mmol, 1eq.) of (**9bb**) and malononitrile (29 mg, 0.4 mmol, 2eq.) were dissolved in 5 ml of chloroform, after 10 min degassing one drop of triethylamine was added and the mixture was refluxed for 1 h. The product was chromatographed on silica gel (eluent petroleum ether/ CH_2_Cl_2_ 8:2) to give 100 mg (91%) of red powder. MS-MALDI m/z = 503.3; ^1^H NMR (CDCl_3_): δ (ppm) = 7.82-7.79 (m, 4H), 7.74 (s, 1H), 7.70 (d, 1H), 7.65-7.56 (m, 6H), 7.45-7.42 (m, 4H), 7.36-7.33 (m, 3H), 7.18 (d, 2H); ^13^C NMR (CDCl_3_): δ (ppm) = 156.8, 150.2, 149.7, 144.2, 140.3, 134.3, 133.2, 130.7, 129.4, 127.6, 127.1, 126.5, 125.5, 125.3, 124.8, 123.4, 122.4, 122.3, 114.5, 113.6.

**Device fabrication**

Indium-tin oxide coated glass slides of 24×25 ×1.1 mm with a surface resistance of 10 Ω/□ were purchased from Kintec company. Part of the ITO layer was etched away with 37% HCl. The ITO electrodes were then cleaned in ultrasonic bath successively with a solution of Deconex (Deconex from VWR international GmbH), distilled water (15.3 MΩ cm^-1^), acetone and ethanol for 10 min each and dried in an oven at 100°C. The electrodes were then modified by a spin-cast layer of PEDOT:PSS (Clevios P VP. AI 4083 (HC-Starck) filtered through a 0.45 µm membrane just prior use). Spin-casting was achieved at 5000 rpm (r = 10 s, t = 60 s), and the electrode was then dried at 110 °C for 15 min. Films of donor materials of 30-35 nm thickness were spun-cast in atmospheric conditions from choloform solutions containing 5 mg mL^-1^. Chloroform (HPLC grade) was distilled over P_2_O_5_ before use. After film deposition the devices were introduced in an argon glovebox (200B, MBraun) equiped with a vacuum chamber and a 30 nm film of C_60_ fullerene (99+%) (MER Corporation) and a 100 nm thick aluminium electrode were thermally evaporated on top of the donor film under a pressure of 2 10^-6^ mbar through a mask defining two cells of 6.0 mm diameter (0.28 cm^2^) on each ITO electrode. C_60_ (99+%) was purshased from MER Corporation and used as received.

The *J* *vs V* curves of the devices were recorded in the dark and under illumination using a Keithley 236 source-measure unit and a home-made acquisition program. The light source was an AM1.5 Solar Constant 575 PV simulator (Steuernagel Lichttecknik, equipped with a metal halogen lamp). The light intensity was measured by a broad-band power meter (13PEM001, Melles Griot). The devices were illuminated through the ITO electrode side. The efficiency values reported here are not corrected for the possible spectral mismatch of the solar simulator. External quantum efficiency (EQE) was measured using a halogen lamp (Osram) with an Action Spectra Pro 150 monochromator, a lock-in amplifier (Perkin-Elmer 7225) and a S2281 photodiode (Hamamatsu).

Hole mobilities have been measured by the space-charge-limited current method on devices ITO/PEDOT:PSS/donor/Au. Donor films of 200-300 nm thickness were spun-cast from solutions of CHCl_3_ (22 mg/mL)(see SI).

**Supplementary information**

Fig.S1.Normalized UV-Vis absorption spectra (3.10^-5^ M in CH_2_Cl_2_)


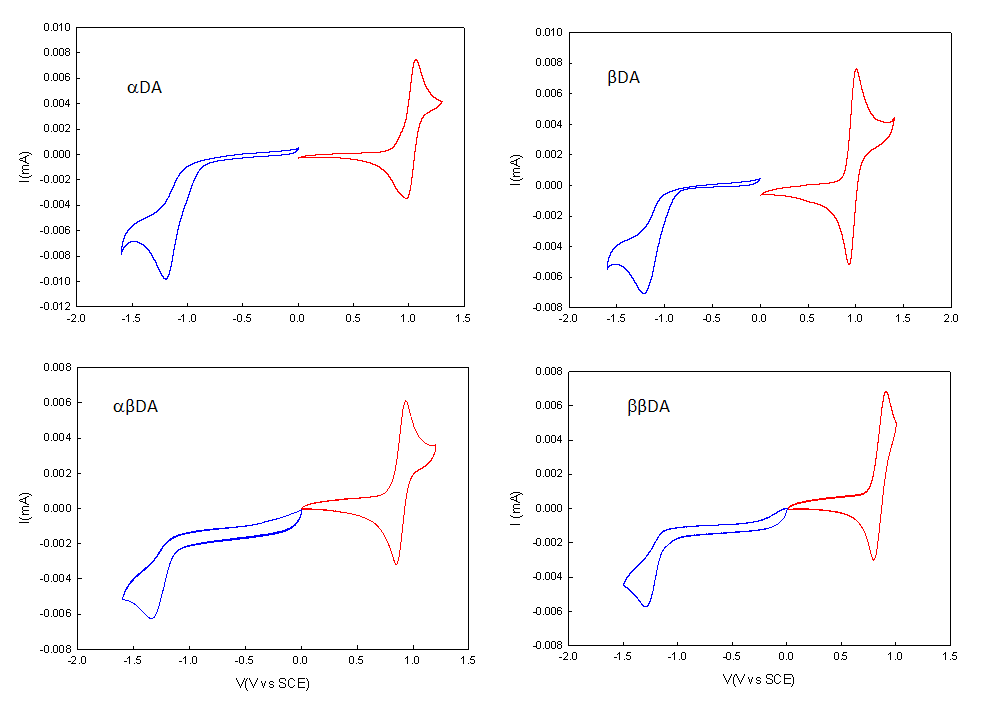


Fig.S2. Cyclic voltammograms of the naphtyl-substituted D-A compounds in 0.10 M Bu_4_NPF_6_/CH_2_Cl_2_, Pt electrodes, v=100mV s^-1^

Table S1. PV results for a series of 4 substrates (8 cells) based on donor 2b

| Cell | V_oc_ (V) | J_sc_ (mA cm^-2^) | FF | η (%) |
| --- | --- | --- | --- | --- |
| 1 | 0.89 | 6.72 | 0.46 | 3.06 |
| 2 | 0.88 | 6.80 | 0.47 | 3.12 |
| 3 | 0.80 | 7.70 | 0.43 | 2.94 |
| 4 | 0.79 | 7.75 | 0.43 | 2.93 |
| 5 | 0.83 | 8.00 | 0.45 | 3.32 |
| 6 | 0.78 | 7.60 | 0.44 | 2.90 |
| 7 | 0.83 | 7.80 | 0.47 | 3.38 |
| 8 | 0.81 | 7.63 | 0.46 | 3.15 |
|  | 0.83 | 7.50 | 0.45 | 3.10±0.20 |

**Determination of hole mobilities**

Hole mobilities have been measured by the space-charge-limited current method on devices ITO/PEDOT:PSS/donor/Au. The donor films were spun-cast from solutions of CHCl_3_ (22 mg/mL) . The thickness of the active layer (L) was **2a** = 214 nm, **2b** d= 277 nm. From the graphs of Fig. S4 the following values were obtained: **2a** *μ* = 4.2x10^-6^ cm² V^-1^s^-1^, **2b** *μ *= 5.5x10^-5^ cm² V^-1^s^-1^.

|  |
| --- |
|  |

**Fig. S4.** Current voltage curves for hole-only devices based on compound **2a** (top) and **2b** (bottom)
